# Sociomoral Temperament: A Mediator Between Wellbeing and Social Outcomes in Young Children

**DOI:** 10.3389/fpsyg.2021.742199

**Published:** 2021-11-08

**Authors:** Darcia Narvaez, Tracy Gleason, Mary Tarsha, Ryan Woodbury, Ying Cheng, Lijuan Wang

**Affiliations:** ^1^Department of Psychology, University of Notre Dame, Notre Dame, IN, United States; ^2^Department of Psychology, Wellesley College, Wellesley, MA, United States

**Keywords:** sociomoral, wellbeing, temperament, self-regulation, social behavior, child development, socialization, triune ethics

## Abstract

Social outcomes, such as empathy, conscience, and behavioral self-regulation, might require a baseline of psychological wellbeing. According to Triune Ethics Metatheory (TEM), early experience influences the neuropsychology underlying a child's orientation toward the social and moral world. Theoretically, a child's wellbeing, fostered through early caregiving, promotes sociomoral temperaments that correspond to the child's experience, such as social approach or withdrawal in face-to-face situations. These temperaments may represent an individual's default sociomoral perspective on the world. We hypothesized that sociomoral temperament emerges as a function of wellbeing and would be related to social outcomes measured by moral socialization and self-regulation. Further, we hypothesized that sociomoral temperament would mediate the relationship between wellbeing and social outcomes. To investigate, we collected items reflective of sociomoral temperament, asking mothers from two countries (USA: *n* = 525; China: *n* = 379) to report on their 3- to 5-year-old children. They also reported on their child's wellbeing (anxiety, depression, happiness) and social outcomes, including moral socialization (concern after wrong doing, internalized conduct and empathy) and behavioral self-regulation (inhibitory control and misbehavior). As expected, correlations identified connections between wellbeing, sociomoral temperament, and social outcomes. Mediation analyses demonstrated that sociomoral temperament mediated relations between wellbeing and social outcomes in both samples, though in slightly different patterns. Fostering early wellbeing may influence social outcomes through a child's developing sociomoral temperament.

## Introduction

Historically, research on morality has focused on cognition, emphasizing the development of moral reasoning and judgment (Kohlberg, 1984; Rest, 1986; Turiel, 2006). More recently, psychologists have started integrating the socio-emotional aspects of moral functioning, such as empathy and social cooperation (Killen and Smetana, 2015), with the cognitive domain (Padilla-Walker and Carlo, 2014), but little work to date has examined the role of psychological wellbeing as a precursor to moral outcomes. This oversight is puzzling given that attending to the needs of others is compromised if individuals are distracted by their own distress (Batson and Oleson, 1991). Wellbeing, including not just the absence of psychopathology but also happiness and thriving, might well be essential for sociomoral capacities, such as empathy and conscience. As a foundation for interacting with the social world, a child's wellbeing theoretically fosters a particular “sociomoral temperament”—an orientation toward others—that either hinders or enables moral social behavior. Given the connections between poor wellbeing and chronic distress (e.g., Lanius et al., 2010), we hypothesized that wellbeing established early in development might be linked to sociomoral temperament, and that individual differences in wellbeing and consequent sociomoral temperament might contribute to variations in moral behavior. If so, intervention efforts designed to promote wellbeing and positive sociomoral temperaments in early childhood might have significant implications for children's social outcomes.

Empirical work supports the notion of a tie between wellbeing and moral outcomes. For example, the significantly compromised wellbeing caused by early toxic stress undermines self-regulatory capacities fundamental for sociality, such as the physiological stress response (Lupien et al., 2009) and vagal tone (Porges, 2011). Similarly, Kochanska and colleagues (e.g., Kochanska, 2002; Kochanska et al., 2005) have written extensively on the Mutually Responsive Orientation (MRO)—the emotional climate of the early parent-child relationship that promotes the development of conscience and the internalization of social mores. While the MRO and wellbeing are not synonymous, a positive emotional environment is a likely component of wellbeing in early childhood. How exactly positive wellbeing connects to outcomes is unclear, so in this work we focused on the details of the implicit systems that result in the “ethical knowhow” (Varela, 1999) necessary for children to exhibit moral behavior. These implicit systems mediating between psychological wellbeing and children's social outcomes is what we are assessing through our measure of sociomoral temperament.

## Wellbeing and Sociomoral Temperament

The idea that wellbeing might have implications for sociomoral temperament has its roots in the relational developmental systems view (Overton, 2013). This perspective suggests that human functioning is not just psychological but deeply embodied, such that a child's wellbeing is a function of neurobiological architecture that shapes implicit assumptions and expectations about social interactions and relationships (Schore, 2019). Animal studies demonstrate that poor wellbeing is associated with cacostatic responses to social situations, such as aggression or withdrawal (Harlow, 1958). We hypothesized that in humans, this link between wellbeing and behavior might be mediated by implicit expectations regarding social interactions, or sociomoral temperament.

Although the relation between wellbeing and sociomoral temperament has not received significant attention in children, for adults, states of wellbeing are related to moral functioning (e.g., Frederickson and Branigan, 2005), and dispositional wellbeing (e.g., agreeableness) predicts prosociality (Meier et al., 2006). Likewise, in adult retrospective reports, Narvaez et al. (2016b) found that poor wellbeing, identified as subclinical psychopathology (depression and anxiety), predicted self-protective sociomoral temperaments emphasizing social opposition or withdrawal, whereas higher wellbeing (a lack of subclinical psychopathology) was associated with a sociomoral temperament characterized by engagement and social approach. We tested whether similar relations could be established as early as the preschool years, given our theory that sociomoral temperament emerges early in development when personality temperament is likewise being formed (Rothbart and Bates, 1998).

## Sociomoral Temperament and Social Outcomes

Our hypothesis that sociomoral temperament would be linked to social outcomes is based in Triune Ethics Meta-theory (TEM; Narvaez, 2008, 2014, 2016). TEM joins the trend toward studying the effects of implicit embodied functioning (rather than cognitive structures only) on psychosocial functioning (Varela et al., 1991), particularly in moral psychology (Narvaez and Lapsley, 2005; Narvaez, 2010). It integrates the interaction of developmentally-relevant experience, neurobiological development, and personality formation to clinical and sub-clinical moral outcomes with the idea that these may help explain the gap between moral judgment and moral action (Lapsley and Narvaez, 2004b). For example, despite having learned and internalized social rules, a person might feel justified to act from fear or rage in the heat of the moment if a social stimulus triggers an intense stress response for protection (e.g., Gilligan, 1997; van der Kolk, 2014).

TEM also holds that neurobiological dispositions are influenced by early wellbeing. For example, the functioning of the hypothalamic–pituitary–adrenal (HPA) system is affected by wellbeing (Lupien et al., 2009), such that with highly stressful experiences, an individual might develop a disposition toward a hyper- or hypo-reactive stress response, possibly undermining capacities for the social attunement required for socioemotional intelligence (Goleman, 1995a,b). Further, early wellbeing is associated with vagus nerve function (vagal tone), which appears fundamental to capacities related to compassionate moral behavior such as social approach and social closeness (Porges, 2011). Experiences that promote physiological and psychological wellbeing may thus influence sociomoral temperament, promoting social *approach* rather than *withdrawal*.

The TEM framework for understanding sociomoral functioning focuses on three basic orientations rooted in global brain states (MacLean, 1990): protectionism, engagement, and imagination. When a particular global brain state or mindset guides behavior, it becomes an ethic. A protectionist orientation emerges from the activation of survival systems (e.g., stress response: fight-flight-freeze-faint; Sapolsky, 2004) and focuses on self-preservation through dominance or withdrawal. The stress response directs perception, thought, and action in self-protective ways. For example, individuals whose neurobiological systems have a low threshold for activation of the stress response are likely to default to protectionist orientations (e.g., perceiving intentional harm when accidentally bumped, Crick and Dodge, 1994). In contrast, an engagement orientation draws on capacities for emotional presence, relational attunement, and unconditional positive regard (Rogers, 1961), which rely on developmental neurobiological capacities like vagal regulation (Porges, 2011) and social oxytocin release (Feldman, 2007). Well-functioning self-regulation, in combination with these neurobiological capacities, enables an engagement orientation. The ethic of imagination is undergirded by abilities to abstract and imagine possibilities outside the present moment. It adds creativity, intentionality, and abstraction into social relations and can be fueled by engagement or protectionist mechanisms.

In adults, triune ethics orientations have been examined with self-report measures that address how much a respondent thinks a list of characteristics represents an orientation they have and how much they think their friends and family would consider the list part of the respondent's characteristics (Narvaez, 2014; Narvaez and Hardy, 2016). Each list of characteristics relates to a particular type of mindset: protectionism (social opposition or withdrawal), engagement, or imagination [one that is generally reflective, detached (withdrawn), vicious (oppositional), or communal]. Although a person can shift among mindsets based on the situation, an adult sociomoral temperament defaulting to self-protectionism generally has been related to a personality pattern of distrust, aggression, and social dominance, as well as less prosocial behavior. In contrast, an engagement temperament has been related to greater agreeableness, conscientiousness, and prosocial behavior variably measured (Narvaez, 2014; Narvaez and Hardy, 2016; Narvaez et al., 2016a).

We tested whether individual differences in sociomoral temperaments would lead to variations in social behavior outcomes in the preschool years. Specifically, we examined variations in aspects of moral socialization, such as empathy and conscience, and in socially-relevant self-regulation, such as inhibitory control and misbehavior. Each of these outcomes is dependent upon a child's ability to regulate internal states sufficiently so as to attend to the requirements of a social situation and the needs of others. A child who has developed a sociomoral temperament of self-protectionism has likely encountered consistent social stress (i.e., associated with compromised wellbeing), which has resulted in behavior that prioritizes safety of the self over the wellbeing of others. Poor wellbeing, associated with repeated stress, could create a default focus on self-preservation (Shanker, 2016) that undermines social self-regulation. As a consequence, regulation in social settings might be compromised, resulting in impulsivity or externalizing behaviors toward others. In contrast, a child whose wellbeing is high will likely have developed the foundations for engagement, allowing for attention to the needs and concerns of others and successful regulation of behavior. Our hypothesis was that early positive wellbeing would be linked to a sociomoral temperament of approach and openness to social experience rather than avoidance or withdrawal. We also expected that sociomoral temperament would connect to social outcomes with implications for moral behavior, such as moral socialization and social self-regulation. Lastly, we expected the relation between wellbeing and outcomes would be mediated by sociomoral temperament.

## Current Studies

**Figure 1:**
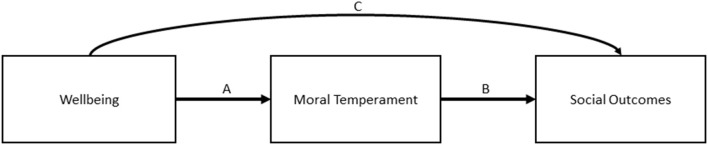
Overall model predicting sociomoral temperament and social outcomes from wellbeing.

Our first goal was to develop a measure of sociomoral temperament, which we named the measure of Child SocioMoral Orientation (CSMO), and to confirm its factor structure. Our second goal was to examine each component of our hypothesized model of the relations between wellbeing, sociomoral temperament, and children's social outcomes (see Figure 1). Given that our overarching hypothesis was that children's wellbeing predicts individual differences in moral socialization and social self-regulation mediated by sociomoral temperament, we tested the relations between (a) our measures of wellbeing (happiness, thriving, anxiety, depression) and the CSMO scores (Figure 1, path A); (b) the CSMO scores and social outcomes (Figure 1, path B), including measures of moral socialization (empathy, concern after wrong doing, internalized conduct) and social self-regulation (inhibitory control, misbehavior); and (c) the extent to which CSMO mediated between wellbeing and social outcomes.

We expected strong positive relationships between wellbeing measures (e.g., thriving) and engagement scores on CSMO, and similarly positive relationships between illbeing (e.g., anxiety), and self-protectionism scores; likewise, we expected illbeing to be negatively correlated with engagement scores, and wellbeing to be negatively correlated with self-protectionism. We also hypothesized that CSMO engagement scores would positively predict social outcomes (socialization and self-regulation), and that self-protectionism would negatively predict these same outcomes. Lastly, we hypothesized that CSMO scores would mediate the relations between wellbeing and child moral outcomes.

We collected large samples in two countries. We selected the USA and China for their historically distinctive cultures, one more individualistic and one more collectivistic (Triandis et al., 1990). We also assessed measurement invariance across the two cultures. Although USA and Chinese cultures differ in several ways, we anticipated that similar outcomes would be found for each sample, including that CSMO would have similar factor structures across cultures because of its focus on fundamental social approach-avoidance, which characterizes human interactions generally. In all our analyses, we controlled for gender and tested for gender differences since they have emerged in past research using some of the measures (e.g., Kochanska, 1994; Clark et al., 2016).

## Study 1

The purposes of this study were to confirm the factor structure of the CSMO measure and assess whether it would mediate the relation between wellbeing and moral outcomes in young children.

### Method

#### Participants

We collected data from US mothers (*n* = 525; *Mage* = 32.97, SD = 5.06 years, range: 20–49; median household income $50,000–$75,000; 84.4% married or in civil union) who reported on their 2- to 4-year-old children (*Mage* = 3.35, SD = 0.46, range 2–5; 295 sons, 229 daughters, 1 “Other”).

#### Procedure

Participants were recruited through fliers at preschools and through electronic notices delivered by parenting-focused organizations, listservs, and blogs. Consent was gathered prior to the start of the survey. Data were collected *via* Qualtrics Survey Software, taking ~45 mins to complete; participants were compensated with a gift card. Study design and data collection procedures were approved by the university's institutional review board.

#### Measures

All measures were parent report. In addition to measuring children's sociomoral temperament through CSMO, we included measures of children's wellbeing as predictors of CSMO, the mediator, and measures of self-regulation and moral socialization as child outcomes.

##### Predictors

*Child Wellbeing*. Child wellbeing was measured *via* four constructs. Five items measured the frequency with which the child demonstrated *happiness* (Gleason et al., 2016; e.g., “Dances spontaneously;” α = 0.72) on a 6-point Likert scale (1 = never to 6 = more than once a day). *Thriving* was assessed using an adapted version (Gleason et al., 2016) of the Warwick-Edinburgh Wellbeing Scale (*n* = 14; e.g., “My child deals well with problems;” α = 0.91) using a 6-point Likert scale (1 = never to 6 = always). We used a 17-item *depression* measure (Gleason et al., 2016; α = 0.92) assessing frequency of depression-related behaviors (e.g., “How often does your child lack confidence?”) using a 6-point Likert scale (1 = never to 6 = several times a day). *Anxiety* was measured using the Preschool Anxiety Scale (Spence et al., 2001; *n* = 29; e.g., “Is afraid of meeting or talking to unfamiliar people,” α = 0.94) rated on a 5-point Likert scale (0 = not true at all to 4 = very often true or not applicable).

##### Mediator

In order to measure sociomoral orientation in children, we ran a pilot study using items from the adult measure of triune ethics orientation (Narvaez and Hardy, 2016; Narvaez et al., 2016a), which measures various forms of self-protectionism (e.g., social withdrawal, social opposition), social engagement, and types of imagination (e.g., reflective, vicious, detached, communal), and adapted them for maternal report of the child in social situations. We added terms to capture children's visible behavior (e.g., freezes, excited). Items (*n* = 71) were randomly presented. Whether the items within the orientations of self-protectionism, engagement, and imagination would fall into factors similar to those in the adult measure was unclear (Narvaez, 2013), so we conducted exploratory factor analyses (EFA) within each of the three orientations (Kline, 2013). Participants included US mothers of preschool children (*N* = 166; 58.9% boys) recruited in the United States through parenting blogs, flyers, a parenting organization, and parenting listservs in the Midwest and Northeast to fill out an online survey in exchange for a gift card. Participants ranged in age from 18 to 48 years (M = 33.56, SD = 5.54). Most mothers (92.0%) were married and 96.9% had at least some college education. Yearly household income varied substantially. And the sample was 82.2% Caucasian Euro-American, with family size ranging from 2-9 people (*M* = 4.43, SD = 1.13), including an average of 2.12 adults (SD = 0.55) and 2.31 children (SD = 0.93).

The extraction method was principal component analysis (PCA) using oblimin rotation because this method is recommended (Kim and Mueller, 1978; Brown, 2009) when factors are expected to correlate. Retention of factors was based on eigenvalues larger than 1 (Kaiser, 1960), inspecting the scree plot (i.e., identifying an elbow), and the variance explained by retained factors.

Eighteen items did not fit into any factor; the remaining 53 items fell into seven factors. Two factors emerged with respect to Social Engagement: (a) Social enjoyment (*n* = 9; α = 0.93) and (b) Social attunement (*n* = 8; α = 0.88). Two factors were associated with Imagination: (c) Social consideration (*n* = 6; α = 0.84) and (d) Social imagination (*n* = 6; α = 0.84). Three factors emerged from the items associated with Self-Protectionism: (e) Social opposition (*n* = 10; α = 0.92), (f) Social distrust (*n* = 4; α = 0.61), and (g) Social withdrawal (*n* = 10; α = 0.91). The seven subscales were then used in the current studies (see Supplemental Materials for factor loadings and notes; the final set of items is listed by factor in the Appendix). We called the final version of the measure Child SocioMoral Orientation (CSMO).

##### Outcomes

*Child Moral Socialization*. Three different aspects of child morality were assessed *via* maternal report using subscales of the My Child survey (Kochanska et al., 1994) which have been demonstrated to correlate with child behavior (Kochanska, 1995): *Empathy* (*n* =13, α = 0.86, “Will try to comfort or reassure another in distress”), *concern after wrongdoing* (*n* = 8, α = 0.88, “When she or he does something wrong, seems to feel relieved when forgiven”) and *internalized conduct* (*n* = 9, α = 0.86, “Clearly hesitates before doing something forbidden, even when alone”). For all scales, mothers rated their children's behavior using a 7-point Likert scale (1 = extremely untrue of your child to 7 = extremely true of your child).

*Child Self-Regulation*. We measured self-regulation using the *inhibitory control* subscale from the Child Behavior Questionnaire (CBQ, Putnam and Rothbart, 2006; *n* = 6; e.g., “Can lower his/her voice when asked to do so”; α = 0.83) rated on a 7-point Likert scale (1 = extremely untrue of your child to 7 = extremely true of your child). We measured frequency of *misbehavior* (Gleason et al., 2016; *n* = 6; e.g., “How often does your child misbehave?” α = 0.75) using a 4-point Likert scale (1 = once a week or less, 2 = several times a week, 3 = every day, 4 = several times a day), except for one question that assessed recent misbehavior (i.e., “How often did your child misbehave in the last week?”) rated on a 5-pt. scale (1 = “not at all,” 5 = “over a dozen times”).

#### Analytic Plan

To investigate factor structure, we used parallel analysis (Horn, 1965) on CSMO subscale scores. After assessing reliability, we ran correlation analyses between CSMO subscale and composite scores with established measures of wellbeing, self-regulation, and moral socialization. Because there were gender differences in prior research with self-regulation and moral socialization measures (i.e., girls scoring higher on inhibitory control, guilt, moral conduct; Kochanska et al., 1997, 2005), we investigated gender differences as well.

EFA analyses were conducted using R Statistical Language (R Core Team, 2016) and add-on packages, such as lavaan (Rosseel, 2012), psych (Revelle, 2016), car (Fox and Weisberg, 2011), Hmisc (Harrell, 2016), and QuantPsyc (Fletcher, 2012). Mediation models were conducted using Mplus (Muthén and Muthén, 2019).

For the EFAs, goodness of fit was assessed using root mean square error of approximation (RMSEA) values below 0.08 and comparative fit index (CFI) values greater than or equal to 0.95 (Hu and Bentler, 1999; Hooper et al., 2008). Chi-square is largely influenced by sample size and considering the number of participants in our studies, CFI and RMSEA were the indices used to evaluate model fit (Shi et al., 2019). However, chi-square was still assessed for all models. In addition, all mediation models are saturated and they have perfect model fit with chi-squares = 0, RMSEA = 0, and CFI =1.

The proportion of missing data differed by variable. In all models, we handled missing data with full-information-maximum-likelihood (FIML), which is suitable for handling data missing completely at random and missing at random (Enders and Bandalos, 2001).

### Results

#### Factor Structure

Parallel analysis identified two factors. The first factor, labeled Self-Protectionism (SP), was measured by Social Opposition, Distrust, and Withdrawal. The second factor, termed Imaginative Relational Attunement (IRA), was measured by Social Enjoyment, Attunement, Imagination, and Consideration. This factor was named IRA because it was a combination of both Engagement and Imagination orientations. Utilizing PROMAX rotation, the two-factor model had good fit and a clean factor structure [robust $\chi_{\left(9\right)}^{2}$ = 32.22, *p* < 0.001, CFI = 0.986, RMSEA = 0.07] and all standardized factor loadings were higher than 0.40 (see Table 1). Further, the two factors were slightly negatively correlated (*r* = −0.12, *p* = 0.04) and corresponded with our notions of sociomoral temperaments connected to social withdrawal and approach. Given that two factors accounted for CSMO items, SP and IRA, the composite scores of these subscales were used in proceeding analyses.

**Table 1:** Study 1 (USA) and Study 2 (China) EFA factor loadings for the child sociomoral orientation measure (CSMO).

	**USA**		**China**	
	**Factor 1**	**Factor 2**	**Factor 1**	**Factor 2**
Opposition	**0.46**	0.03	**0.57**	0.05
Distrust	**0.77**	0.03	**0.82**	−0.01
Withdrawal	**0.77**	−0.02	**0.62**	−0.03
Attunement	0.03	**0.93**	0.00	**0.84**
Consideration	0.02	**0.87**	0.06	**0.84**
Imagination	0.00	**0.77**	−0.01	**0.81**
Enjoyment	−0.07	**0.79**	−0.07	**0.79**

*Bold values show significant loadings, p < 0.05*.

#### Descriptives and Correlations

Table 2 presents descriptive statistics. In this study, the proportion of missing data ranged from 2.7 to 9.6%, and sample size ranges are listed in under USA for boys, girls, and the total sample. After correcting for multiple tests, three gender differences emerged: girls had significantly higher means than boys on happiness *t*_(457.18)_ = −4.42, *p* < 0.001, empathy, *t*_(503.39)_ = −3.74, *p* < 0.001, and inhibitory control, *t*_(482.62)_ = −5.14, *p* < 0.001. Because of these differences, we used gender as a control variable in our models.

**Table 2:** Descriptive statistics for Study 1 (USA) and Study 2 (China) by country and gender with between-group t-tests.

	**USA**				**China**				**Between-country comparisons**		
	**Boys** **(*n* = 253-295)**	**Girls** **(*n* = 213-229)**	**Total** **(*n* = 475-511)**	**Between-gender *difference* for USA**	**Boys** **(*n* = 179-188)**	**Girls** **(*n* = 185-191)**	**Total** **(*n* = 365-380)**	**Between-gender *difference* for China**	**Boys**	**Girls**	**Total**
	***M* (SD)**	***M* (SD)**	***M* (SD)**	***t* (Cohen's d)**	***M* (SD)**	***M* (SD)**	***M* (SD)**	***t* (Cohen's *d*)**	***t* (Cohen's *d*)**	***t* (Cohen's *d*)**	***t* (Cohen's *d*)**
**Wellbeing**											
Happiness	5.24 (0.69)	5.49(0.52)	5.35 (0.63)	−4.42^*^ (−0.40)	4.39 (0.95)	4.54(0.96)	4.47 (0.96)	−1.52 (−0.16)	10.38^*^ (1.06)	12.01^*^ (1.26)	15.42^*^ (1.12)
Thriving	5.37 (0.55)	5.50(0.45)	5.43 (0.51)	−2.82 (−0.25)	4.73 (0.74)	4.73(0.78)	4.73 (0.76)	−0.03 (0.00)	10.01^*^ (1.01)	11.85^*^ (1.23)	15.30^*^ (1.11)
Depression	2.08 (0.70)	1.90(0.64)	1.99 (0.68)	2.90 (0.26)	1.77 (0.66)	1.63(0.64)	1.70 (0.65)	2.03 (0.22)	4.69^*^ (0.45)	4.02^*^ (0.42)	6.38^*^ (0.43)
Anxiety	1.49 (0.53)	1.42(0.48)	1.46 (0.51)	1.48 (0.14)	2.38 (0.88)	2.27(0.94)	2.32 (0.91)	1.19 (0.12)	−12.00^*^ (−1.29)	−10.84^*^ (−1.17)	−16.01^*^ (−1.21)
**Sociomoral temperament**											
CSMO subscales											
Opposition	2.72 (0.99)	2.51 (0.94)	2.63 (0.98)	2.37 (0.21)	2.07 (0.83)	1.89 (0.69)	1.98 (0.77)	2.32 (0.23)	7.56^*^ (0.70)	7.61^*^ (0.74)	10.90^*^ (0.73)
Distrust	2.12 (0.92)	2.02 (0.87)	2.07 (0.90)	1.17 (0.11)	1.73 (0.88)	1.59 (0.77)	1.66 (0.83)	1.52 (0.17)	4.56^*^ (0.43)	5.21^*^ (0.52)	6.96^*^ (0.47)
Withdrawal	2.44 (0.80)	2.42 (0.80)	2.43 (0.80)	0.26 (0.025)	2.16 (0.75)	2.15 (0.72)	2.15 (0.73)	0.09 (0.01)	3.83^*^ (0.36)	3.57^*^ (0.35)	5.26^*^ (0.36)
Attunement	5.09 (0.72)	5.25 (0.71)	5.16 (0.72)	−2.52 (0.22)	3.90 (1.06)	3.86 (1.21)	3.88 (1.14)	0.41 (0.04)	13.23^*^ (1.36)	13.85^*^ (1.43)	19.07^*^ (1.38)
Consideration	4.92 (0.80)	5.06 (0.73)	5.00 (0.77)	−1.73 (−0.18)	3.71 (1.18)	3.55 (1.07)	3.64 (1.13)	1.37 (0.14)	12.22^*^ (1.25)	16.06^*^ (1.67)	19.75^*^ (1.44)
Imagination	4.87 (0.83)	5.09 (0.83)	4.97 (0.84)	−2.89 (−0.26)	3.90 (1.18)	3.79 (1.17)	3.87 (1.17)	1.11 (0.09)	9.32^*^ (0.98)	12.57^*^ (1.30)	15.37^*^ (1.10)
Enjoyment	5.58 (0.60)	5.65 (0.73)	5.61 (0.58)	−1.35 (−0.12)	4.72 (1.14)	4.62 (1.17)	4.67 (1.15)	0.78 (0.09)	9.45^*^ (1.00)	10.98^*^ (1.08)	14.43^*^ (1.08)
CSMO factor scores											
Self-protectionism	2.43 (0.73)	2.32 (0.41)	2.38 (0.71)	1.69 (0.15)	1.99 (0.65)	1.88 (0.59)	1.93 (0.62)	1.65 (0.18)	14.53^*^ (0.63)	15.81^*^ (0.88)	9.75^*^ (0.67)
IRA	5.12 (0.65)	5.26 (0.63)	5.18 (0.64)	−2.47 (−0.22)	4.07 (0.98)	3.96 (1.00)	4.02 (0.99)	0.87 (0.11)	7.09^*^ (1.31)	11.03^*^ (1.58)	12.55^*^ (1.43)
**Social outcomes**											
Moral socialization											
Empathy	5.22 (0.61)	5.42 (0.58)	5.31 (0.60)	−3.74^*^ (−0.33)	5.00 (0.75)	5.09 (0.60)	5.05 (0.68)	−1.35 (−0.13)	3.43^*^ (0.33)	5.57^*^ (0.56)	6.02^*^ (0.41)
Concern after wrongdoing	5.06 (1.17)	5.28 (1.18)	5.16 (1.18)	−1.99 (−0.19)	5.20 (0.98)	5.22 (0.91)	5.21 (0.94)	−0.22 (−0.02)	−1.37 (−0.13)	0.50 (0.06)	−0.67 (−0.05)
Internalized conduct	4.24 (1.08)	4.42 (1.11)	4.32 (1.09)	−1.87 (−0.17)	4.10 (0.55)	4.15 (0.49)	4.13 (0.52)	−1.03 (−0.10)	1.85 (0.15)	3.25^*^ (0.31)	3.42^*^ (0.21)
Self-regulation											
Inhibitory control	4.79 (0.85)	5.18 (0.81)	4.97 (0.85)	−5.14^*^ (−0.46)	4.64 (0.80)	4.86 (0.78)	4.75 (0.79)	−2.62 (−0.28)	1.95 (0.18)	4.12^*^ (0.40)	3.96^*^ (0.27)
Misbehavior	2.75 (0.50)	2.63 (0.41)	2.69 (0.47)	3.00 (0.26)	2.28 (0.50)	2.20 (0.34)	2.24 (0.45)	1.67 (0.18)	9.32^*^ (0.94)	10.40^*^ (1.13)	13.80^*^ (0.98)

*CSMO, child sociomoral orientation; IRA, imaginative relational attunement*.

*T-tests were performed with a Bonferroni correction (c = 18)*.

* *p < 0.002778*.

Table 3 contains the correlation coefficients (above diagonal). As predicted, positive correlations emerged between wellbeing and IRA and between illbeing and SP. The reverse was also mostly supported, although SP did not correlate with happiness. The fact that SP did not correlate with happiness, whereas IRA did, suggests the importance of treating IRA and SP separately. Generally, these findings are consistent with the idea that psychological systems are associated with differentiated sociomoral orientations.

**Table 3:** Within country pearson's correlations of all variables for Study 1 (USA) and Study 2 (China).

	**1**	**2**	**3**	**4**	**5**	**6**	**7**	**8**	**9**	**10**	**11**	**12**	**13**	**14**	**15**	**16**	**17**	**18**	**19**
**Wellbeing**																			
1. Happiness	–	0.56^**^	−0.29^**^	−0.26^**^	0.55^**^	0.52^**^	0.46^**^	0.48^**^	0.48^**^	−0.01	−0.05	0.15^**^	−0.12^**^	0.37^**^	0.21^**^	0.11^*^	0.23^**^	−0.04	0.20^**^
2. Thriving	0.46^**^	–	−0.46^**^	−0.40^**^	0.58^**^	0.54^**^	0.51^**^	0.50^**^	0.52^**^	−0.18^**^	−0.19^**^	0.09	−0.29^**^	0.50^**^	0.22^**^	0.13^**^	0.31^**^	−0.19^**^	0.13^**^
3. Depression	−0.10^*^	−0.25^**^	–	0.40^**^	−0.26^**^	−0.24^**^	−0.23^**^	−0.20^**^	−0.25^**^	0.54^**^	0.52^**^	0.21^**^	0.47^**^	−0.28^**^	0.00	−0.21^**^	−0.30^**^	0.43^**^	−0.14
4. Anxiety	0.01	−0.14^**^	0.50^**^	–	−0.27^**^	−0.24^**^	−0.20^**^	−0.23^**^	−0.31^**^	0.37^**^	0.17^**^	0.20^**^	0.48^**^	−0.17^**^	0.01	−0.03	−0.12^*^	0.17^**^	−0.07
**CSMO**																			
5. IRA (comp.)	0.37^**^	0.43^**^	−0.12^*^	0.08	–	0.92^**^	0.89^**^	0.87^**^	0.86^**^	0.03	−0.02	0.24^**^	−0.14^**^	0.55^**^	0.22^**^	0.22^**^	0.42^**^	−0.16^**^	0.12^*^
6. Attunement	0.36^**^	0.38^**^	−0.10	−0.08	0.56^**^	–	0.82^**^	0.69^**^	0.74^**^	0.03	−0.05	0.23^**^	−0.10^*^	0.60^**^	0.26^**^	0.25^**^	0.41^**^	−0.18^**^	0.12^**^
7. Consideration	0.31^**^	0.34^**^	−0.05	0.05	0.72^**^	0.66^**^	–	0.66^**^	0.67^**^	−0.00	−0.16^**^	0.25^**^	−0.07	0.53^**^	0.27^**^	0.36^**^	0.52^**^	−0.27^**^	0.08
8. Imagination	0.32^**^	0.44^**^	−0.08	−0.06	0.55^**^	0.64^**^	0.69^**^	–	0.69^**^	0.07	0.06	0.23^**^	−0.14^**^	0.41^**^	0.11^*^	0.09	0.27^**^	−0.06	0.14^**^
9. Enjoyment	0.35^**^	0.40^**^	−0.16^**^	−0.13^*^	0.53^**^	0.70^**^	0.60^**^	0.63^**^	–	0.01	0.09	0.13^**^	−0.21^**^	0.40^**^	0.12	0.07	0.26^**^	−0.05	0.06
10. SP (comp.)	−0.02	−0.15^**^	0.52^**^	0.28^**^	−0.03	0.03	0.08	0.03	−0.02	–	0.74^**^	0.74^**^	0.79^**^	−0.11^*^	0.06	−0.14^**^	−0.17^**^	0.40^**^	−0.05
11. Opposition	−0.06	−0.07	0.45^**^	0.18^**^	−0.07	−0.01	0.04	0.11^*^	0.05	0.77^**^	–	0.25^**^	0.36^**^	−0.22^**^	−0.10^*^	−0.37^**^	−0.40^**^	0.60^**^	−0.11^*^
12. Distrust	0.01	−0.07	0.37^**^	0.22^**^	0.05	0.07	0.13^*^	0.05	−0.01	0.84^**^	0.48^**^	–	0.49^**^	0.13^**^	0.18^**^	0.08	0.13^**^	0.06	0.02
13. Withdrawal	−0.00	−0.21^**^	0.44^**^	0.28^**^	−0.04	0.02	0.03	−0.09	−0.09	0.78^**^	0.36^**^	0.53^**^	–	−0.15^**^	0.08	0.02	−0.06	0.18^**^	−0.02
**Child outcomes**																			
14. Empathy	0.29^**^	0.30^**^	−0.16^**^	0.03	0.72^**^	0.26^**^	0.22^**^	0.18^**^	0.27^**^	−0.06	−0.08	−0.01	−0.07	–	0.29^**^	0.27^**^	0.44^**^	−0.28^**^	0.16^**^
15. Concern	0.22^**^	0.31^**^	−0.09	0.07	0.76^**^	0.21^**^	0.18^**^	0.22^**^	0.21^**^	−0.12^*^	−0.15^**^	−0.04	−0.10	0.55^**^	–	0.19^**^	0.21^**^	−0.07	0.10^*^
16. Int. conduct	0.08	0.14^**^	−0.04	0.10	0.23^**^	0.06	0.12^*^	0.03	0.03	−0.02	−0.09	0.00	0.06	0.32^**^	0.15^**^	–	0.62^**^	−0.40^**^	0.08
17. Inh. control	0.15^**^	0.28^**^	0.17^**^	−0.00	0.54^**^	0.31^**^	0.33^**^	0.22^**^	0.25^**^	−0.14^**^	−0.28^**^	−0.01	−0.06	0.43^**^	0.45^**^	0.31^**^	–	−0.41^**^	0.23^**^
18. Misbehavior	−0.05	−0.01	0.20^**^	0.03	−0.09	−0.07	−0.13^*^	0.02	0.01	0.32^**^	0.45^**^	0.19^**^	0.07	−0.02	−0.00	−0.16^**^	−0.22^**^	–	−0.15^**^
19. Gender	0.04	−0.01	−0.11^**^	−0.06	−0.02	−0.02	−0.08	−0.06	−0.04	−0.10	−0.12^*^	−0.12^*^	−0.01	0.07	0.01	0.05	0.14^**^	−0.07	–

*IRA (comp.), Imaginative Relational Attunement (composite), SP (comp.), self-protectionism (composite)*.

*USA and China correlation coefficients are above and below the diagonal respectively*.

* *p < 0.0*;

** *p < 0.01*.

Most correlations between CSMO subscales and the self-regulation and moral socialization measures were in the expected directions. The results for the self-protectionism subscales were mixed. Most correlations were negative, as hypothesized, except that distrust correlated positively with empathy, concern after wrongdoing, and inhibitory control. Correlations were not significant between social distrust and internalized conduct and misbehavior, nor did social withdrawal correlate with concern, internalized conduct, or inhibitory control.

#### Contributions of Sociomoral Temperament When Mediating the Relationship Between Wellbeing and Child Outcomes

**Figure 2:**
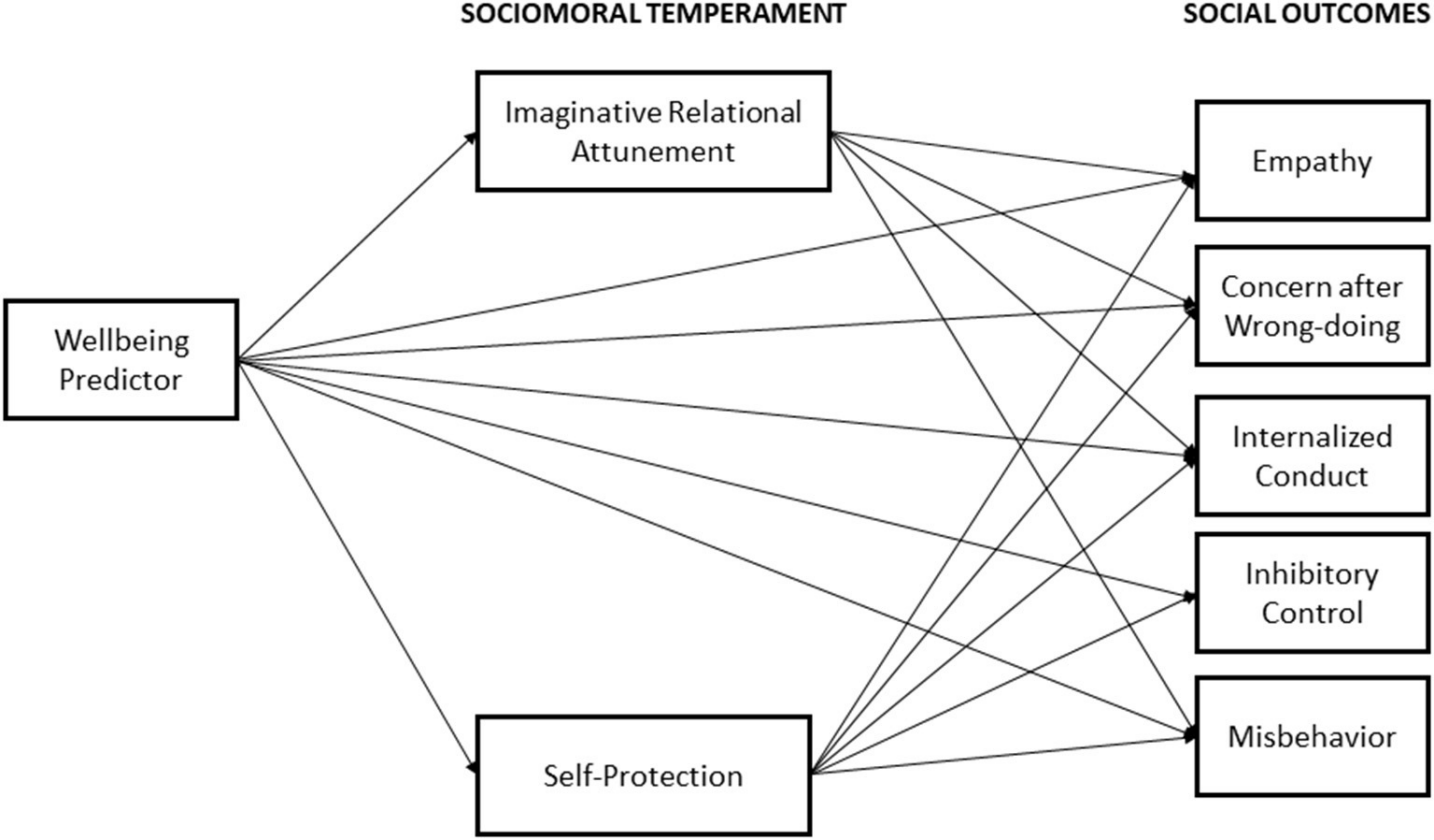
Mediation model with moral temperament mediating relation between wellbeing and social outcomes.

We next tested the hypothesis that CSMO composite scores would mediate the relations between wellbeing and child social outcomes (Figure 1, path C). We constructed four models, each of which used a wellbeing measure (happiness, thriving, depression, and anxiety) as a predictor, IRA and SP as mediators, and the moral socialization (empathy, concern after wrong doing, internalized conduct) and self-regulation (inhibitory control, misbehavior) measures as outcomes (see Figure 2 for the mediation model and Table 4 for model results). All child outcomes were allowed to correlate, yielding four saturated, perfect fitting models, $\chi_{\left(0\right)}^{2}$ = 1.00, *p* = 0.00, CFI = 1.00, RMSEA = 0.00. Gender was included as a control variable.

**Table 4:** Study 1 (USA) summary of mediation effects.

**Predictor outcome**	**â: path to IRA (*p*)**	**b: path from IRA (*p*)**	**â: path to SP (*p*)**	**b: path from SP (*p*)**	â ^*^ b IRA [95% CI]	â ^*^ b SP [95% CI]
Happiness	2.18 (<0.001)		0.00 (1.00)			
Empathy		0.12 (<0.001)		−0.04 (0.001)	0.26 (0.20, 0.32)	0.00 (−0.02, 0.01)
Concern		0.60 (0.009)		0.03 (0.234)	0.14 (0.03, 0.26)	0.00 (−0.01, 0.02)
Int. conduct		0.10 (<0.001)		−0.08 (0.001)	0.22 (0.12, 0.34)	0.00 (−0.03, 0.02)
Inh. control		0.14 (<0.001)		−0.08 (<0.001)	0.30 (0.22, 0.39)	0.00 (−0.03, 0.02)
Misbehavior		−0.05 (<0.001)		0.11 (<0.001)	−0.16 (−0.13, −0.05)	0.00 (−0.01, 0.05)
Thriving	2.85 (<0.001)		−0.69 (<0.001)			
Empathy		0.10 (<0.001)		−0.03 (0.025)	0.27 (0.20, 0.36)	0.02 (0.00, 0.04)
Concern		0.05 (0.035)		0.05 (0.087)	0.16 (0.00, 0.32)	−0.03 (−0.09, 0.01)
Int. conduct		0.10 (<0.001)		−0.09 (0.001)	0.29 (0.17, 0.42)	0.06 (0.02, 0.11)
Inh. control		0.12 (<0.001)		−0.07 (<0.001)	0.35 (0.26, 0.45)	0.05 (0.02, 0.09)
Misbehavior		−0.03 (0.002)		0.11 (<0.001)	−0.09 (−0.15, −0.04)	−0.07 (−0.12, −0.03)
Depression	−0.99 (<0.001)		0.05 (0.740)			
Empathy		0.12 (<0.001)		−0.03 (0.063)	−0.11 (−0.16, −0.06)	−0.04 (−0.09, 0.01)
Concern		0.10 (<0.001)		0.01 (0.744)	−0.09 (−0.15, −0.04)	0.02 (−0.10, 0.13)
Int. conduct		0.08 (<0.001)		−0.05 (0.119)	−0.07 (−0.12, −0.04)	−0.07 (−0.16, 0.01)
Inh. control		0.12 (<0.001)		−0.05 (0.018)	−0.11 (−0.16, −0.06)	−0.08 (−0.14, −0.01)
Misbehavior		−0.02 (0.029)		0.07 (<0.001)	0.02 (0.00, 0.04)	0.20 (0.12, 0.28)
Anxiety	−1.22 (<0.001)		1.39 (<0.001)			
Empathy		0.16 (<0.001)		−0.04 (0.005)	−0.15 (−0.24, −0.08)	−0.05 (−0.09, −0.01)
Concern		0.10 (<0.001)		0.03 (0.373)	−0.12 (−0.24, −0.04)	0.04 (−0.06, 0.13)
Int. conduct		0.12 (<0.001)		−0.10 (<0.001)	−0.12 (−0.22, −0.07)	−0.14 (−0.25, −0.06)
Inh. control		0.14 (<0.001)		−0.09 (<0.001)	−0.12 (−0.27, −0.08)	−0.12 (−0.18, −0.07)
Misbehavior		−0.03 (<0.001)		0.11 (<0.001)	0.04 (0.02, 0.07)	0.15 (0.10, 0.22)

*IRA, imaginative relational attunement; SP, self-protectionism; CI, confidence interval*.

*The confidence intervals were bootstrapped. The formula is: point estimate ± z*

* *s/√n*.

Mediation analyses tested both total (i.e., both mediators together) and specific (i.e., each CSMO mediator) indirect mediation effects of wellbeing on child outcomes. For specific indirect effects, IRA significantly mediated the relationships from all four wellbeing measures to all five outcomes at *p*s ≤ 0.002 with two caveats: the strengths of the specific indirect paths from thriving to concern and depression to misbehavior were lower but still significant (*p* ≤ 0.045). For SP, the results were more mixed. None of the specific indirect effects of SP were significant when mediating happiness and the five outcomes. However, SP mediated the relationship from thriving and depression to three outcomes: internalized conduct and inhibitory control (*p*s ≤ 0.04), and misbehavior (*p*s ≤ 0.006). SP also mediated paths between anxiety and all outcomes except concern (*p*s ≤ 0.008).

Regarding direct effects, happiness predicted concern after wrong-doing (*p* = 0.03) and, contrary to our hypotheses, positively predicted misbehavior (*p* = 0.02). Thriving directly predicted empathy and concern (*p* ≤ 0.009), whereas depression predicted inhibitory control (*p* = 0.02) and misbehavior (*p* < 0.001). Both depression and anxiety predicted internalized conduct (*p* ≤ 0.045).

### Discussion

These findings suggest that fostering wellbeing may be a significant contribution to future moral socialization and self-regulation *via* sociomoral temperament. Specifically, the connections between our measures of wellbeing and moral socialization were successfully mediated in whole or in part by our measure of sociomoral temperament. The relations were stronger for the self-regulatory outcomes, in that those predicted by thriving and anxiety were not accompanied by direct effects. These stronger relations to self-regulatory than moral socialization outcomes might be a function of development, in that the former might emerge earlier than the latter (Berger et al., 2007). Also, as the relation between physiological wellbeing and self-regulation is well-established (Lupien et al., 2009), these results suggest that one mechanism of that connection in early childhood might be sociomoral temperament.

## Study 2

Our goal in Study 2 was to test whether these models applied equally well in a different culture. Despite the significant cultural variation between the USA and China, we expected CSMO to have a similar factor structure because of its focus on fundamental social approach-avoidance, which characterizes human interactions generally. This second sample afforded examination of measurement invariance across cultures as well as group comparisons with the USA data. We also conducted the same examination of mediation effects of sociomoral temperament in relation to wellbeing and sociomoral outcomes as in Study 1.

### Method

#### Participants

Data were collected from Chinese mothers (*n* = 382; *Mage* = 33.83 years; range: 21–45; median income $120,000–$160,000 US; 95% of mothers married or in civil unions). They were recruited through six Beijing preschools (Child *Mage* = 4.48, *SD* =0.90; 188 girls, 191 boys, 3 missing). Institutional review board approval and consent for participation were gathered before the survey was started. Participants received a parenting book to compensate them for their time.

#### Procedure

Chinese mothers responded *via* paper and pencil to all the same measures as USA mothers in Study 1.

#### Measures

Most measures had been translated and validated in Chinese in a previous study (Narvaez et al., 2013). The remainder was translated into Mandarin (including standard procedures for back translation).

*Child Wellbeing*. The same measures were used as in Study 1. Happiness had an alpha of 0.62; thriving α = 0.81; depression α = 0.86; anxiety α = 0.90.

*Child Moral Socialization*. The same measures were used as in Study 1. Empathy had an alpha of 0.80; concern after wrongdoing α = 0.78; internalized conduct α = 0.60.

*Child Self-Regulation*. The same measures were used as in Study 1. Inhibitory control had an alpha of 0.81; misbehavior α = 0.67.

#### Analytical Plan

We conducted a confirmatory factor analysis. In order to be able to compare country means, we tested for measurement invariance.

### Results

#### Factor Structure and Measurement Invariance

We detected a similar factor structure as in the USA data. We found that a two-factor CFA model acceptably fit the data [robust $\chi_{\left(11\right)}^{2}$ = 31.66, *p* < 0.001, CFI = 0.98, RMSEA = 0.07] and all standardized factor loadings were higher than 0.50 (see Table 1). Self-Protectionism and Imaginative Relational Attunement were not significantly correlated.

After confirming the two-factor structure for the CSMO within China, we tested for measurement invariance between the USA and China at the scale level. We obtained partial invariance by freeing two parameters (factor loadings of “social enjoyment” and “social opposition” intercepts) and constraining all other factor loadings and manifest variable intercepts to be equal across countries (model 3b). Model 3b was the final model and suggested partial strong invariance (see Table 5). With such a model, we were able to compare the means of the two factors between countries.

**Table 5:** Goodness-of-fit indices and Chi-square difference tests for models testing measurement invariance with respect to country.

	**Model compared**	**Chi-square**	**df**	**Chi. Dif**.	**df Dif**.	***p*-value**
1. Baseline (configural)		210.15	26	–	–	–
2a. Invariant loadings	1	235.20	31	19.27	5	0.002
2b. Invariant loadings (free: Λ_enjoyment_)	1	214.16	30	3.54	4	0.472
3a. Invariant loadings and intercepts (free: Λ_enjoyment_)	2b	269.59	35	44.68	5	<0.001
3b. Invariant loadings and intercepts (free: Λ_enjoyment_; υ_opposition_)	2b	226.08	34	8.93	4	0.063
4. Invariant loadings, intercepts, and residuals (free: Λ_enjoyment_; υ_opposition_)	3b	693.35	41	374.85	7	<0.001

*Λ = factor loading; υ = manifest intercept*.

Table 2 includes comparisons on each scale between and within country and gender. In this study, missing data ranged from <1 to 4% and sample ranges are listed under China for boys, girls, the total sample. We also investigated whether the factor score differences by country were linked to specific CSMO sub-scales. For all CSMO subscales, USA children scored significantly higher than Chinese children, even after a Bonferroni correction. No gender differences in CSMO scales emerged within country. However, we wondered whether CSMO subscale differences between countries were related to gender; the lack of correlations between countries suggested that this difference may not be related to gender. USA boys and girls scored significantly higher than their Chinese counterparts on all CSMO scales and subscales.^1^

Correlations are presented in Table 3 (below diagonal). The hypothesis that wellbeing would be positively correlated with engagement scores and ill-being with self-protectionism was supported (Figure 1, path A), but the reverse was not well-supported. Depression was only negatively correlated with IRA and enjoyment, and anxiety only with the latter; thriving was only negatively correlated with SP and withdrawal. However, these few negative correlations underscore the idea that engagement and self-protection are not exclusive of one another but that an individual can display one or the other in different social situations (Narvaez, 2014), which a social-cognitive theory would predict (Lapsley and Narvaez, 2004a).

Similar to the USA, we found partial support for the hypothesis that CSMO engagement scores and social outcomes of self-regulation and moral socialization would be positively correlated (Figure 1, path B). Few negative correlations emerged between CSMO Self-Protectionism scores and child outcomes, although they were positively correlated (as expected) with misbehavior. In general, IRA predicted some aspects of moral socialization, but only misbehavior was linked to SP. Withdrawal in particular was unrelated to any child outcomes, suggesting that Chinese mothers did not associate fearfulness with moral socialization or self-regulation.

#### Contributions of Sociomoral Temperament When Mediating the Relationship Between Wellbeing and Child Outcomes

We tested for mediation effects using the same model as the USA sample (Figure 1, path C), which also yielded four saturated, perfect fitting models, $\chi_{\left(0\right)}^{2}$ = 1.00, *p* = 0.00, CFI = 1.00, RMSEA = 0.00 (see Table 6). For specific indirect effects, IRA significantly mediated the same relationships as the total indirect effects for happiness and thriving (*p*s ≤ 0.002). For depression, IRA significantly mediated the relationship with empathy and inhibitory control (*p*s = 0.03). IRA did not yield any significant specific indirect effects for anxiety. SP did not mediate relationships between happiness, thriving, and the five outcome variables. However, SP significantly mediated the relationship between depression, anxiety and concern after wrong-doing (*p*s ≤ 0.005) and between anxiety and inhibitory control (*p* = 0.004).

**Table 6:** Study 2 (China) summary of mediation effects.

**Predictor outcome**	**â: path to IRA (*p*)**	**b: path from IRA (*p*)**	**â: path to SP (*p*)**	**b: path from SP (*p*)**	â ^*^ b IRA [95% CI]	â ^*^ b SP (95% CI)
Happiness	1.29 (<0.001)		−0.03 (0.737)			
Empathy		0.04 (<0.001)		−0.02 (0.374)	0.05 (0.02, 0.09)	0.00 (−0.01, 0.01)
Concern		−0.01 (0.797)		−0.13 (0.001)	−0.01 (−0.10, 0.08)	0.003 (−0.03, 0.02)
Int. conduct		0.01 (0.451)		−0.01 (0.727)	0.01 (−0.01, 0.02)	0.00 (−0.03, 0.02)
Inh. control		0.07 (<0.001)		0.002 (0.738)	0.07 (0.05, 0.14)	−0.06 (−0.02, 0.01)
Misbehavior		−0.02 (<0.001)		0.00 (0.965)	−0.02 (−0.06, −0.001)	0.00 (−0.003, 0.003)
Thriving	2.33 (<0.001)		−0.23 (0.050)			
Empathy		0.03 (0.002)		−0.01 (0.650)	0.03 (0.02, 0.14)	−0.01 (−0.01, 0.01)
Concern		0.03 (0.159)		−0.14 (<0.001)	0.07 (−0.01, 0.17)	0.03 (−0.001, −0.08)
Int. conduct		0.00 (0.997)		−0.00 (0.959)	0.00 (−0.04, 0.04)	−0.00 (−0.01, 0.01)
Inh. control		0.05 (<0.001)		−0.05 (0.008)	0.13 (0.07, 0.21)	0.01 (−0.003, 0.03)
Misbehavior		−0.01 (0.343)		−0.01 (0.532)	−0.01 (−0.03, 0.004)	0.001 (−0.01, 0.009)
Depression	−0.73 (0.020)		1.44 (<0.001)			
Empathy		0.05 (<0.001)		0.01 (0.818)	−0.03 (−0.07,−0.008)	0.01 (−0.04, 0.07)
Concern		0.04 (0.012)		−0.12 (0.002)	−0.03 (−0.08, 0.003)	−0.17 (−0.34, −0.04)
Int. conduct		0.01 (0.221)		−0.00 (0.807)	0.01 (−0.02, 0.01)	−0.00 (−0.06, 0.05)
Inh. control		0.07 (<0.001)		−0.04 (0.066)	−0.05 (−0.01, −0.12)	−0.06 (−0.14, 0.00)
Misbehavior		−0.01 (0.010)		0.00 (0.899)	0.01 (.00, 0.02)	0.002 (−0.05, 0.04)
Anxiety	−0.18 (0.430)		0.60 (<0.001)			
Empathy		0.05 (<0.001)		−0.02 (0.225)	−0.01 (−0.03, 0.01)	−0.01 (−0.04, 0.01)
Concern		0.02 (0.324)		−0.13 (0.001)	−0.08 (−0.03, 0.01)	−0.08 (−0.14, −0.35)
Int. conduct		0.01 (0.150)		−0.02 (0.251)	−0.01 (−0.01, 0.004)	0.07 (−0.03, 0.01)
Inh. control		0.07 (<0.001)		−0.07 (0.001)	−0.01 (−0.05, 0.02)	−0.04 (−0.08, −0.02)
Misbehavior		−0.02 (<0.001)		0.01 (0.628)	0.00 (−0.01, 0.02)	0.04 (−0.09, 0.01)

*IRA, Imaginative Relational Attunement; SP, self-protectionism; CI, confidence interval*.

*The confidence intervals were bootstrapped. The formula is: point estimate ± z*

* *s/√n*.

Direct effects emerged for happiness in relation to empathy and concern after wrong-doing (*p*s ≤ 0.001). Thriving likewise directly predicted all but concern (*p*s ≤ 0.02). Depression directly predicted empathy (*p* = 0.02) and anxiety directly predicted internalized conduct (*p* = 0.02).

## Discussion

In this sample, a two-factor structure for CSMO again emerged, and sociomoral temperament mediated some of the relations between wellbeing and social outcomes. The mediation analyses suggest the greatest role for sociomoral temperament was between the illbeing predictors (depression and anxiety) and concern and inhibitory control, at least with respect to total indirect effects. Similar patterns emerged for total indirect and IRA-specific mediation between happiness and inhibitory control and misbehavior. In comparison to the USA sample, these findings suggest possible cultural differences with respect to the role of sociomoral temperament in connecting wellbeing and social outcomes.

Most of the CSMO differences between subscales that emerged were country rather than gender differences, with the USA having significantly higher scores even after adjustments for multiple comparisons. For the subscales associated with IRA, these findings are consistent with other research indicating that American mothers tend to emphasize their children's successes and deemphasize their failures, whereas Chinese mothers do the opposite (Ng et al., 2007). Although CSMO factors are not measuring successes and failures per se, the generally positive valence of the items might have elicited higher endorsement from American mothers than Chinese mothers. Additionally, if expectations about the appropriateness of displaying fear, anger, or timidity differ between cultures, what children show and how parents rate those behaviors might also differ (Louie et al., 2013). At the same time, the Chinese mothers scored their children lower on every CSMO factor subscale except anxiety. Chinese culture is a shame-socialized culture, emphasizing maintenance of others' approval, especially elders (filial piety), and avoidance of disappointing others (Schoenhals, 1993); consequently, parenting even of young children tends to emphasize right behavior, compliance after wrongdoing and making amends (Fung, 1999). Perhaps low scores in China occurred because Chinese citizens tend to minimize emotions in their lives (Ryder et al., 2008), and they have specific longstanding etiquette rules for behavior such as self-control and obedience to elders, which they may consequently judge more harshly (Conrad, 2019).

## General Discussion

In these studies, we hypothesized that sociomoral temperament would mediate the relationship between wellbeing and sociomoral outcomes in two countries, the USA and China. As hypothesized, we found a two-factor solution for our measure of sociomoral temperament in both samples for Imaginative Relational Attunement (IRA) and Self-Protectionism (SP), and in both samples, mediation analyses demonstrated that these factors influenced the relationship between wellbeing and social outcomes. These findings support the theory that psychological wellbeing influences moral development through children's orientation toward the social world in early childhood, though results need to be understood in light of their cross-sectional nature.

Similarities and differences emerged in the patterns when examining models across countries. For example, IRA mediated everything in the USA but not in China. With respect to child outcomes, at least one factor of sociomoral temperament effectively mediated the relationship with the wellbeing measures in the USA, and connections emerged for all outcomes except internalized conduct for China. For internalized conduct, the Chinese scores were less varied than in the US sample, which might account for the lack of mediation, and further exploration of parents' conceptualization of internalized conduct across cultures would illuminate these relationships at this point in development.

Across both countries IRA demonstrated a higher number of specific and direct effects than SP. One interpretation of this result is that an engaged sociomoral temperament plays a greater and more varied role in mediating between wellbeing and social outcomes than does self-protectionism. However, the samples were drawn from typically-developing, middle-class populations that scored higher on thriving and happiness and lower on anxiety and depression. They exhibited more variation on the IRA than the SP scores. A wider range of scores on SP, such as that obtainable in clinical populations, might result in greater variation. In typically-developing samples like these, who have higher happiness and thriving scores, IRA might be a more typical outcome. Thus, as a positive mediator, IRA might have captured more variance than SP. This idea does not negate SP as a mediator but does suggest a need for exploration in samples that have less attenuated scores.

### The Importance of Wellbeing for Sociomoral Temperament, Moral Socialization, and Self-Regulation

Our analyses demonstrated relations between wellbeing, self-regulation, and moral socialization with many relations mediated by CSMO scales in the USA and fewer in China. These results have several implications for understanding the development of morality in early life and possible directions for future research, including the embodied nature of moral functioning, the developmental progression of capacities relevant to moral behavior, and the usefulness of sociomoral temperament as a construct and component of moral development.

Theoretically, both wellbeing and CSMO orientations are based in biosocial functioning. Understanding the psychobiological mechanisms that lead to individual variations in wellbeing, and concomitantly, sociomoral temperament, is a promising avenue for future research. For example, parental responsivity, especially given its relation to moral socialization measures (Kochanska, 2002), is likely predictive of the enhanced wellbeing that would foster development of an IRA orientation. However, other experiences, such as those with direct links to biosocial processes such as self-regulation, might have significant influence on the neurological underpinnings of a self-protectionist versus a relational attunement social orientation (Narvaez, 2014).

The fact that sociomoral temperament mediated the relations between wellbeing and self-regulation more so than moral socialization—and that these findings emerged in both samples (albeit in different patterns)—might be explained by the point in development at which these processes were measured (Berger et al., 2007). Children's development of empathy and concern after wrongdoing, for example, requires a significant cognitive component that includes attention to another's feelings—a capacity that is not fully formed in early childhood. Inhibitory control and the prevention of misbehavior, in contrast, are self-focused behaviors influenced by social contexts. These behaviors are components of moral development because of their implications for interpersonal relationships, but their mastery, depending as it does on curbing impulsivity, might be experienced intrapersonally at least some of the time, meaning that children might confront, practice, and master these developmental tasks prior to those that involve the perspectives of others. If so, the implication of these findings is that at this point in early childhood, wellbeing might be particularly important for promoting self-regulation both directly and through the construction of the implicit systems measured by sociomoral temperament. Whether these relations emerge similarly at other points in development is a topic for future research.

Mediating effects of sociomoral temperament across both countries also point to the possible explanatory power of TEM. Regardless of cultural influences, sociomoral temperament, as understood through social approach (IRA) and social withdrawal (SP) temperaments, helps explain the relationship between wellbeing and child social outcomes in early childhood. Similar to the TEM adult studies (Narvaez, 2014; Narvaez and Hardy, 2016; Narvaez et al., 2016a), evidence from this study suggests that TEM global temperaments are important variables in understanding the links between wellbeing and social outcomes. Our results contribute to this growing literature but also extend it by providing evidence that sociomoral temperament is important for understanding *child* wellbeing and social outcomes. The mediation model suggests that the exclusion of sociomoral temperament may lead to misrepresentation of the relationship between child wellbeing and social outcomes. For both research and practice, including sociomoral temperament may be necessary to both adequately investigate and understand the way in which child wellbeing relates to social outcomes.

### Limitations and Future Directions

This paper has several limitations. First, it was a cross-sectional set of studies which allow only a glimpse into potential developmental trajectories. Second, the data were collected in different ways, online across the USA in one sample and on paper in several preschools in China. These strategies may have had an effect on the nature of who was recruited and how they responded. Third, the correlations found between our measures of sociomoral temperament and other child outcomes might have been inflated by the use of maternal report for both. Observational measures should be used in future studies. Fourth, while the comparison of the USA and China is useful, truly establishing cultural invariance of CSMO requires more samples. Fifth, we did not include tests of the childhood environment so we cannot relate CSMO scores to childhood experiences (although see Tarsha et al., 2020). Future work might use CSMO to assess relationships between aspects of wellbeing and changes or stability in sociomoral temperament over time. Such an approach might illustrate whether the timing and intensity of experiences related to wellbeing are critical to the development of a more flexible and open sociomoral temperament.

Despite these limitations, our results do provide some support for the role of sociomoral temperaments in children's social outcomes. According to TEM, these different sociomoral orientations are based upon global brain states or neurobiological dispositions influenced by wellbeing. Future research should consider taking into consideration the components of wellbeing that promote or deter social approach or withdrawal.

## Conclusion

The findings presented here support the idea that early wellbeing influences social outcomes and that sociomoral temperament helps explain this relationship. Our results suggest that sociomoral temperament is a mechanism worth investigating in explaining the connection between psychological wellbeing and young children's moral development. The pattern of findings between the USA and China samples suggests that some relation between wellbeing, sociomoral temperament, and social outcomes might be universal, but that differences in cultural expectations, perhaps with respect to children's obedience to adult authority (Fung, 1999), might govern the exact connections that emerge. As children's wellbeing helps formulate children's sociomoral temperament, the inclination to approach or withdraw from social interaction, the ways in which they learn to function in the world appear to have significance for later social capacities. Viewing morality as a function of holistic wellbeing might have significant ramifications for understanding how psychological functioning influences children's nascent morality and consequent social functioning.

## Data Availability Statement

The raw data supporting the conclusions of this article will be made available by the authors, without undue reservation.

## Ethics Statement

The studies involving human participants were reviewed and approved by University of Notre Dame Institutional Review Board. The patients/participants provided their written informed consent to participate in this study.

## Author Contributions

All authors listed have made a substantial, direct and intellectual contribution to the work, and approved it for publication.

## Funding

We thank the Institute for Scholarship in the Liberal Arts at the University of Notre Dame for its financial support.

## Conflict of Interest

The authors declare that the research was conducted in the absence of any commercial or financial relationships that could be construed as a potential conflict of interest.

## Publisher's Note

All claims expressed in this article are solely those of the authors and do not necessarily represent those of their affiliated organizations, or those of the publisher, the editors and the reviewers. Any product that may be evaluated in this article, or claim that may be made by its manufacturer, is not guaranteed or endorsed by the publisher.

## Footnotes

^1^When the same item is given to different groups of respondents, they may read it differently owing to cultural differences, which is why we examined measurement invariance across countries. We did not examine gender invariance as the items were given to mothers of small children, instead of children themselves, and we did not expect child gender to influence a mother's reading or interpretation of our items.

## Appendix

### Child Sociomoral Orientation Measure (CSMO)

Think of your child in SOCIAL SITUATIONS. Indicate how much your child shows the following behaviors. Response scale: I have never seen my child be this way or heard that my child acts this way.

I have seen my child be this way or heard that my child acts this way a couple of times ever.

I have seen my child be this way or heard that my child acts this way multiple times in the past.

I have seen my child be this way or heard that my child acts this way every week.

I have seen my child be this way or heard that my child acts this way every day.

I have seen my child be this way or heard that my child acts this way several times a day.

**Table:** Ethic of Self-Protection.

**Social opposition**	**Social distrust**	**Social withdrawal**
Combative	Watchful	Timid
Easily upset	Suspicious	Withdrawing
Hostile	Untrusting	Anxious
Argumentative	Vigilant	Cowardly
Uncooperative		Fearful
Aggressive		Nervous
Fights easily		Scared
Angry		Hesitant
Threatening		Wallflower
Hot-tempered		Freezes

**Table:** Ethic of Engagement.

**Social enjoyment**	**Social attunement**	**Social consideration**
Excited	Forgiving	Thoughtful
Laughs	Gentle	Attentive
Happy	Kind hearted	Considerate of others
Pleasant	Cuddly	Moral
Cheerful	Sympathetic	Honorable
Loving	Empathic	Respectful
Affectionate	Supportive	
Playful	Comforting	
Cheerfully interactive		

### Ethic of Imagination

Social Imagination

Creative

Original

Enterprising

Thinks of new ideas

Artistic

Innovative

## References

[B1] Batson C. D. Oleson K. C. (1991). Current status of the empathy-altruism hypothesis, in Prosocial Behavior, ed Clark M. S. (Sage Publications, Inc.), 62–85.

[B2] Berger A. Kofman O. Livneh U. Henik A. (2007). Multidisciplinary perspectives on attention and the development of self-regulation. Prog. Neurobiol. 82, 256–286. 10.1016/j.pneurobio.2007.06.004 17651888

[B3] Brown J.D. (2009). Choosing the Right Type of Rotation in PCA and EFA. Shiken: JALT Testing and Evaluation SIG Newsletter.

[B4] Clark D. A. Listro C. J. Lo S. L. Durbin C. E. Donnellan M. B. Neppl T. K. (2016). Measurement invariance and child temperament: an evaluation of sex and informant differences on the Child Behavior Questionnaire. Psychol. Assess. 28:1646. 10.1037/pas0000299 26914022 PMC4999341

[B5] Conrad R. (2019). Culture Hacks: Deciphering Differences in American, Chinese, and Japanese Thinking. New York, NY: Lioncrest.

[B6] Crick N. R. Dodge K. A. (1994). A review and reformulation of social information processing mechanisms in children's social adjustment. Psychol. Bull. 115, 74–101. 10.1037/0033-2909.115.1.74

[B7] Enders C. K. Bandalos D. L. (2001). The relative performance of full information maximum likelihood estimation for missing data in structural equation models. Struct. Equ. Model. 8, 430–457. 10.1207/S15328007SEM0803_5

[B8] Feldman R. (2007). Parent-infant synchrony: biological foundations and developmental outcomes. Curr. Direct. Psychol. Sci. 16, 340–345. 10.1111/j.1467-8721.2007.00532.x

[B9] Fletcher T. D. (2012). QuantPsyc: Quantitative Psychology Tools (Version 1.5) [R package]. Available online at: https://CRAN.R-project.org/package=QuantPsyc (accessed June 15, 2015).

[B10] Fox J. Weisberg S. (2011). An R Companion to Applied Regression (2nd Edn.). Thousand Oaks, CA: Sage.

[B11] Frederickson B. L. Branigan C. (2005). Positive emotions broaden the scope of attention and thought-action repertoires. Cogn. Emot. 19, 313–332. 10.1080/02699930441000238 21852891 PMC3156609

[B12] Fung H. (1999). Becoming a moral child: the socialization of shame among young Chinese children. Ethos 27, 180–209. 10.1525/eth.1999.27.2.180

[B13] Gilligan J. (1997). Violence: Reflections on a National Epidemic. New York, NY: Vintage.

[B14] Gleason T. Narvaez D. Cheng Y. Wang L. Brooks J. (2016). Well-being and sociomoral development in preschoolers: the role of maternal parenting attitudes consistent with the evolved developmental niche, in Contexts for Young Child Flourishing: Evolution, Family and Society, eds. Narvaez D. Braungart-Rieker J. Miller L. Gettler L. Hastings P. (New York, NY: Oxford University Press), 166–184. 10.1093/acprof:oso/9780190237790.003.0008

[B15] Goleman D. (1995a). Social Intelligence: The New Science of Human Relationships. New York, NY: Arrow.

[B16] Goleman D. (1995b). Emotional Intelligence: Why it Can Matter More Than IQ. New York, NY: Bantam Books.

[B17] Harlow H. (1958). The nature of love. Am. Psychol. 13, 673–685. 10.1037/h0047884 4984312

[B18] Harrell F. E. Jr. (2016). Hmisc: Harrell Miscellaneous (Version 4.0-2) [R package]. Available online at: https://CRAN.R-project.org/package=Hmisc (accessed June 15, 2015).

[B19] Hooper D. Coughlan J. Mullen M. R. (2008). Structural equation modelling: guidelines for determining model fit. Electron. J. Bus. Res. Methods 6, 53–60. 10.21427/D7CF7R

[B20] Horn J. L. (1965). A rationale and test for the number of factors in factor analysis. Psychometrika 30, 179–185. 10.1007/BF02289447 14306381

[B21] Hu L. T. Bentler P. M. (1999). Cutoff criteria for fit indexes in covariance structure analysis: conventional criteria versus new alternatives. Struct. Equ. Model. 6, 1–55. 10.1080/10705519909540118

[B22] Kaiser H. F. (1960). The application of electronic computers to factor analysis. Educ. Psychol. Meas. 20, 141–151. 10.1177/001316446002000116

[B23] Killen M. Smetana J. G. (2015). Origins and development of morality, in Handbook of Child Psychology and Developmental Science: Socioemotional Processes, eds Lamb M. E. Lerner R. M. (Hoboken, NJ: Wiley), 701–749.

[B24] Kim J. O. Mueller C. W. (1978). Introduction to Factor Analysis. Beverly Hills, CA: Sage. 10.4135/9781412984652

[B25] Kline R. B. (2013). Exploratory and confirmatory factor analysis, in Applied Quantitative Analysis in the Social Sciences, eds Petscher Y. Schatschneider C. (New York, NY: Routledge), 171–207.

[B26] Kochanska G. (1994). Beyond cognition: expanding the search for the early roots of internalization and conscience. Dev. Psychol. 30, 20–22.

[B27] Kochanska G. (1995). Children's temperament, mothers' discipline, and security of attachment: multiple pathways to emerging internalization. Child Dev. 66, 597–615. 10.2307/1131937

[B28] Kochanska G. (2002). Mutually responsive orientation between mothers and their young children: a context for the early development of conscience. Curr. Dir. Psychol. Sci. 11, 191–195. 10.1111/1467-8721.00198

[B29] Kochanska G. DeVet K. Goldman M. Murray K. Putnam S. (1994). Maternal reports of conscience development and temperament in young children. Child Dev. 65, 852–869. 10.2307/1131423 8045172

[B30] Kochanska G. Forman D. R. Aksan N. Dunbar S. B. (2005). Pathways to conscience: early mother–child mutually responsive orientation and children's moral emotion, conduct, and cognition. J. Child Psychol. Psychiatry 46, 19–34. 10.1111/j.1469-7610.2004.00348.x 15660641

[B31] Kochanska G. Murray K. Coy K. C. (1997). Inhibitory control as a contributor to conscience in childhood: from toddler to early school age. Child Dev. 68, 263–277. 10.2307/1131849 9180001

[B32] Kohlberg L. (1984). Essays on Moral Development, Volume 2: The Psychology of Moral Development. San Francisco: Harper and Row.

[B33] Lanius R. A. Vermetten E. Pain C. (2010). The Impact of Early Life Trauma on Health and Disease: The Hidden Epidemic. New York, NY: Cambridge University Press.

[B34] Lapsley D.K. Narvaez D. (eds.) (2004b). Moral Development, Self and Identity: Essays in Honor of Augusto Blasi. Mahwah, NJ: Erlbaum.

[B35] Lapsley D. K. Narvaez D. (2004a). A social-cognitive view of moral character, in Moral Development: Self and Identity, eds Lapsley D. K. Narvaez D. (Mahwah, NJ: Erlbaum), 189–212.

[B36] Louie J. Y. Oh B. J. Lau A. S. (2013). Cultural differences in the links between parental control and children's emotional expressivity. Cult. Diver. Ethnic Minor. Psychol. 19, 424–434. 10.1037/a0032820 23834255

[B37] Lupien S. J. McEwen B. S. Gunnar M. R. Heim C. (2009). Effects of stress throughout the lifespan on the brain, behaviour and cognition. Nat. Rev. Neurosci. 10, 434–445. 10.1038/nrn2639 19401723

[B38] MacLean P. D. (1990). The Triune Brain in Evolution: Role in Paleocerebral Functions. New York, NY: Plenum. 10.1126/science.250.4978.303-a 17797318

[B39] Meier B. P. Robinson M. D. Wilkowski B. M. (2006). Turning the other cheek: agreeableness and the regulation of aggression-related primes. Psychol. Sci. 17, 136–142. 10.1111/j.1467-9280.2006.01676.x 16466421

[B40] Muthén L. K. Muthén B. (2019). Mplus. The Comprehensive Modelling Program for Applied Researchers: User's Guide 5. Los Angeles, CA: Muthén & Muthén

[B41] Narvaez D. (2008). Triune ethics: the neurobiological roots of our multiple moralities. New Ideas Psychol. 26, 95–119. 10.1016/j.newideapsych.2007.07.008

[B42] Narvaez D. (2010). The emotional foundations of high moral intelligence, in Children's Moral Emotions and Moral Cognition: Developmental and Educational Perspectives. New Directions for Child and Adolescent Development, vol 129, eds Latzko B. Malti T. (San Francisco: Jossey-Bass), 77–94. 10.1002/cd.276 20872605

[B43] Narvaez D. (2013). Neurobiology and moral mindsets, in Moral and Immoral Behavior: Theoretical and Empirical Perspectives on Moral Motivation, eds Heinrichs K. Oser F. (Rotterdam: Sense Publishers), 289–307. 10.1007/978-94-6209-275-4_19

[B44] Narvaez D. (2014). Neurobiology and the Development of Human Morality: Evolution, Culture and Wisdom. New York, NY: W.W. Norton.

[B45] Narvaez D. (2016). Embodied Morality: Protectionism, Engagement and Imagination. New York, NY: Palgrave-Macmillan.

[B46] Narvaez D. Hardy S. (2016). Measuring triune ethics orientations, in Embodied Morality: Protectionism, Engagement and Imagination, ed Narvaez D. (New York: Palgrave-Macmillan), 47–72.

[B47] Narvaez D. Lapsley D. (2005). The psychological foundations of everyday morality and moral expertise, in Character Psychology and Character Education, eds Lapsley D. Power C. (Notre Dame, IN: University of Notre Dame Press), 140–165.

[B48] Narvaez D. Thiel A. Kurth A. Renfus K. (2016a). Past moral action and ethical orientation, in Embodied Morality: Protectionism, Engagement and Imagination, ed Narvaez D. (New York, NY: Palgrave-Macmillan), 99–118.

[B49] Narvaez D. Wang L. Cheng A. (2016b). Evolved developmental niche history: relation to adult psychopathology and morality. Appl. Dev. Sci. 4, 294–309. 10.1080/10888691.2015.1128835

[B50] Narvaez D. Wang L. Gleason T. Cheng Y. Lefever J. Deng L. (2013). The evolved developmental niche and sociomoral outcomes in Chinese 3-year-olds. Eur. J. Dev. Psychol. 10, 106–127.

[B51] Ng F. F.-Y. Pomerantz E. Lam S. (2007). European American and Chinese parents' responses to children's success and failure: implications for children's responses. Dev. Psychol. 43, 1239–1255. 10.1037/0012-1649.43.5.1239 17723048

[B52] Overton W. F. (2013). A new paradigm for developmental science: relationism and relational-developmental-systems. Appl. Dev. Sci. 17, 94–107. 10.1080/10888691.2013.778717 23834001

[B53] Padilla-Walker L. M. Carlo G. (eds.) (2014). Prosocial Development: A Multidimensional Approach. New York, NY: Oxford University Press.

[B54] Porges S. (2011). Polyvagal Theory. New York, NY: Norton.

[B55] Putnam S. P. Rothbart M. K. (2006). Development of short and very short forms of the children's behavior questionnaire. J. Personal. Assess. 87, 103–113. 10.1207/s15327752jpa,8701_09 16856791

[B56] R Core Team (2016). R: A LANGUAGE and Environment for Statistical Computing. Vienna: R Foundation for Statistical Computing. Available online at: http://www.R-project.org (accessed June 15, 2015).

[B57] Rest J. R. (1986). Moral Development: Advances in Research and Theory. New York, NY: Praeger Press.

[B58] Revelle W. (2016). p*sych: Procedures for Personality and Psychological Research (Version 1.6.12)* [R package]. Available online at: https://CRAN.R-project.org/package=psych (accessed June 15, 2015).

[B59] Rogers C. (1961). On Becoming a Person: A Therapist's View of Psychotherapy. London: Constable.

[B60] Rosseel Y. (2012). lavaan: an R package for structural equation modeling. J. Stat. Softw. 48, 1–36. 10.18637/jss.v048.i0225601849

[B61] Rothbart M. K. Bates J. E. (1998). Temperament, in Handbook of Child Psychology: Vol. 3. Social, Emotional, and Personality Development, ed Eisenberg N. (New York, NY: Wiley), 105–176.

[B62] Ryder A. G. Yang J. Zhu X. Yao S. Yi J. Heine S. J. Bagby R. M. (2008). The cultural shaping of depression: somatic symptoms in China, psychological symptoms in North America? J. Abnorm. Psychol. 117, 300–313. 10.1037/0021-843X.117.2.300 18489206

[B63] Sapolsky R. (2004). Why Zebras Don't Get Ulcers, 3rd Edn. New York, NY: Holt.

[B64] Schoenhals M. (1993). The Paradox of Power in a People's Republic of China Middle School. New York, NY: Routledge.

[B65] Schore A. N. (2019). The Development of the Unconscious Mind. New York, NY: W.W. Norton.

[B66] Shanker S. (2016). Self-Reg: How to Help Your Child (and You) Break the Stress Cycle and Successfully Engage With Life. Toronto: Penguin Canada.

[B67] Shi D. Lee T. Maydeu-Olivares A. (2019). Understanding the model size effect on SEM fit indices. Educ. Psychol. Meas. 79, 310–334. 10.1177/0013164418783530 30911195 PMC6425088

[B68] Spence S. H. Rapee R. McDonald C. Ingram M. (2001). The structure of anxiety symptoms among preschoolers. Behav. Res. Therapy 39, 1293–1316. 10.1016/S0005-7967(00)00098-X 11686265

[B69] Tarsha M. S. Narvaez D. Gleason T. (2020). Children's Play Is Associated with Wellbeing, Autonomic Regulation and Sociomoral Development. Chicago, IL: Association for Psychological Science.

[B70] Triandis H. McCusker C. Hui C. (1990). Multimethod probes of individualism and collectivism. J. Personal. Soc. Psychol. 59, 1006–1020. 10.1037/0022-3514.59.5.1006

[B71] Turiel E. (2006). The development of morality (revised edition), in Handbook of Child Psychology (Sixth Edition), Volume 3, eds Damon W. Lerner R. M.. New York, NY: Wiley.

[B72] van der Kolk B. (2014). The Body Keeps the Score. New York: Penguin.

[B73] Varela F. (1999). Ethical Know-How: Action, Wisdom, and Cognition. Stanford, CA: Stanford University Press.

[B74] Varela F. J. Thompson E. Rosch E. (1991). The Embodied Mind: Cognitive Science and Human Experience. Cambridge, MA: MIT Press.

