# Supplementary material for: Sociomoral Temperament: A Mediator Between Wellbeing and Social Outcomes in Young Children
Source: Front Psychol. 2021 Nov 8;12:742199. doi: 10.3389/fpsyg.2021.742199 (PMC8606405; doi:10.3389/fpsyg.2021.742199)
Supplement: Supplementary file 1 [file Data_Sheet_1.docx]

Supplemental Material: Exploratory Factor Analysis of the Draft

Child Socio-Moral Orientation Measure

In order to measure sociomoral orientation in children, we adapted items from the adult measure of triune ethics (Narvaez & Hardy 2016), adding items appropriate to children’s behavior, for a total of 71 items, grouped into three subtypes: Self-protectionism, Engagement, and Imagination. We were not sure if the items within these ethics would fall into factors similar to those in the adult measure (Narvaez & Hardy 2016), so we conducted exploratory factor analyses (EFA) within each of the ethics.

Participants included mothers of 3- to 5-year-olds (*N* = 166; 58.9% boys) recruited in the United States through parenting blogs, flyers, a parenting organization, and parenting listservs in the Midwest and Northeast to fill out an online survey in exchange for a gift card. Participants ranged in age from 18 to 48 years (M = 33.56, SD = 5.54). Most mothers (92.0%) were married and 96.9% had at least some college education. Yearly household income varied substantially. And the sample was 82.2% Caucasian Euro-American, with family size ranging from 2-9 people (M = 4.43, SD = 1.13), including an average of 2.12 adults (SD = .55) and 2.31 children (SD = .93).

**Self-Protectionism**

According to Triune Ethics Meta-Theory (Narvaez, 2013), self-protectionism can be either aggressive or withdrawing, which we call bunker security or wallflower security respectively. Consequently, we conducted our factor analyses for self-protectionism within these two categories.

**EFA of Bunker Security items.** Eighteen items related to bunker security (α = .91). EFA with Varimax rotation revealed two factors from these items based on visual inspection of the scree plot as well as the percentage of variance explained (~50%). Fourteen items loaded on a general aggression factor. We ran a factor analysis on these 14 items, and items with factor loadings lower than .4 were dropped. This led to a 10-item subscale of “social opposition” (α = .92). Vigilant, watchful, suspicious, and untrusting loaded on the second factor, “social distrust” (α = .61; see Table A1 in which factor loadings < .4 were suppressed; see Tables A2 and A3 for descriptive statistics for each subscale).

Table A1.

*Factor Loading Matrix of Bunker Security Items*

|  | Factor | |
| --- | --- | --- |
|  | 1 | 2 |
| Combative | .720 |  |
| Easily upset | .679 |  |
| Hostile | .663 |  |
| Argumentative | .712 |  |
| Reactive | .472 |  |
| Vigilant |  | .573 |
| Uncooperative | .613 |  |
| Domineering | .532 |  |
| Bossy | .651 |  |
| Watchful |  | .583 |
| Aggressive | .807 |  |
| Fights easily | .809 |  |
| Challenges others | .614 |  |
| Suspicious |  | .745 |
| Untrusting |  | .711 |
| Angry | .700 |  |
| Threatens | .606 |  |
| Hot-tempered | .810 |  |

Table A2

*Item Statistics for the Social Opposition Subscale*

|  | *Mean* | *Standard Deviation* |
| --- | --- | --- |
| Combative | 2.70 | 1.512 |
| Easily upset | 3.53 | 1.328 |
| Hostile | 2.19 | 1.202 |
| Argumentative | 3.50 | 1.441 |
| Uncooperative | 3.35 | 1.306 |
| Aggressive | 2.55 | 1.337 |
| Fights easily | 2.70 | 1.496 |
| Angry | 3.46 | 1.270 |
| Threatens | 2.01 | 1.257 |
| Hot-tempered | 2.98 | 1.481 |

Table A3

*Item Statistics for Items of the Distrust Subscale*

|  | *Mean* | *Standard Deviation* |
| --- | --- | --- |
| Vigilant | 3.80 | 1.484 |
| Watchful | 4.08 | 1.432 |
| Suspicious | 2.18 | 1.209 |
| Untrusting | 2.16 | 1.172 |

**EFA of Wallflower Security items.** There were 15 items related to wallflower security (α = .91). EFA suggested that this group of items were largely unidimensional (over 45% of variance was explained by one factor). One item, “unwilling” had a factor loading lower than .4, and was the first item dropped. Subsequently when the EFA was rerun, items with low factor loadings were dropped, including “submissive”, “hides”, “refuses to communicate” and “does not interact”. The final subscale “social withdrawal” had 10 items (α = .91). Item statistics are provided in Table A4.

Table A4

*Item Statistics for Items of the Social Withdrawal Subscale*

|  | *Mean* | *Standard Deviation* |
| --- | --- | --- |
| Timid | 2.90 | 1.136 |
| Withdraws | 2.25 | 1.270 |
| Anxious | 2.75 | 1.241 |
| Cowardly | 2.24 | 1.181 |
| Fearful | 2.62 | 1.145 |
| Nervous | 2.73 | 1.199 |
| Scared | 2.83 | 1.034 |
| Hesitant | 3.16 | 1.238 |
| Wallflower | 2.50 | 1.300 |
| Freezes | 2.01 | 1.260 |

**EFA of Engagement**

There were 22 items related to engagement (α = .95). The EFA and factor loading matrix suggested two factors, together accounting for ~60% of the variance. The first factor, “social enjoyment”, included items such as laughs, excited, happy, pleasant, cheerful, loving, affectionate, playful, and cheerfully interactive (α = .93). The second factor, “social attunement”, included 8 items such as forgiving, gentle, kind hearted, cuddly, supportive, comforting, sympathetic and empathic (α =.88). Factor loading matrix and item statistics for these two subscales are provided in Tables A5 - A7.

Table A5

*Factor Loading Matrix of Engagement Items*

|  | Factor | |
| --- | --- | --- |
|  | 1 | 2 |
| Laughs | .835 |  |
| Forgiving | .752 |  |
| Gentle | .873 |  |
| Excited | .613 |  |
| Kind hearted | .770 |  |
| Cuddly | .761 |  |
| Happy | .612 |  |
| Pleasant | .777 |  |
| Cheerful | .674 |  |
| Loving |  | .554 |
| Supportive |  | .612 |
| Comforting |  | .662 |
| Affectionate |  | .515 |
| Playful |  | .758 |
| Cheerfully interactive |  | .721 |
| Sympathetic |  | .805 |
| Empathic |  | .805 |

Table A6

*Item Statistics for Items of the Social Enjoyment Subscale*

|  | *Mean* | *Standard Deviation* |
| --- | --- | --- |
| Laughs | 5.73 | .640 |
| Excited | 5.44 | .789 |
| Happy | 5.71 | .627 |
| Pleasant | 5.57 | .668 |
| Cheerful | 5.55 | .790 |
| Loving | 5.59 | .778 |
| Affectionate | 5.60 | .770 |
| Playful | 5.65 | .718 |
| Cheerfully interactive | 5.51 | .822 |

Table A7

*Item Statistics for Items of the Social Attunement Subscale*

|  | *Mean* | *Standard Deviation* |
| --- | --- | --- |
| Forgiving | 4.89 | .985 |
| Gentle | 5.21 | .897 |
| Kind hearted | 5.40 | .809 |
| Cuddly | 5.38 | 1.036 |
| Supportive | 4.96 | .990 |
| Comforting | 5.05 | 1.030 |
| Sympathetic | 4.91 | 1.033 |
| Empathic | 4.81 | 1.119 |

**EFA of Imagination**

Sixteen items related to imagination (α = .88). EFA and factor loading matrix suggest two factors, a “social imagination” factor formed by items including creative, thinks of new ideas, artistic, enterprising, original, and innovative; and the other factor, “social consideration”, formed by items including thoughtful, attentive, considerate of others, moral, honorable, and respectful. The two factors together accounted for around 48% of the total variance. Items “inclusive” and “fanciful” did not load on either factor and were dropped. The item “imaginative” cross-loaded and was also dropped. When these items were dropped, rerunning the factor analysis the item “reflective” did not load on either factor and was further dropped (see Table A8). This resulted in a 6-item subscale of “social imagination” (α = .84) and a 6-item subscale “social consideration” (α = .84). See Tables A9 and A10 for descriptive statistics.

Table A8

*Factor Loading Matrix of All Imagination Items*

|  | Component | |
| --- | --- | --- |
|  | 1 | 2 |
| Creative | .674 |  |
| Thoughtful |  | .716 |
| Reflective | .462 |  |
| Inclusive |  |  |
| Attentive |  | .634 |
| Thinks of new ideas | .639 |  |
| Considerate of others |  | .814 |
| Fanciful |  |  |
| Imaginative | .545 | .458 |
| Artistic | .649 |  |
| Moral |  | .692 |
| Honorable |  | .561 |
| Enterprising | .607 |  |
| Original | .678 |  |
| Innovative | .811 |  |
| Respectful |  | .773 |

Table A9

*Item Statistics for Items of the Social Imagination Subscale*

|  | *Mean* | *Standard Deviation* |
| --- | --- | --- |
| Creative | 5.28 | .982 |
| Thinks of new ideas | 5.14 | 1.005 |
| Artistic | 4.78 | 1.162 |
| Enterprising | 4.27 | 1.410 |
| Original | 4.89 | 1.052 |
| Innovative | 4.87 | 1.200 |

Table A10

*Item Statistics for Items of the Social Consideration Subscale*

|  | *Mean* | *Standard Deviation* |
| --- | --- | --- |
| Thoughtful | 5.24 | .850 |
| Attentive | 5.17 | .905 |
| Considerate of others | 5.18 | .828 |
| Moral | 4.90 | .970 |
| Honorable | 4.71 | 1.075 |
| Respectful | 5.16 | .912 |
